# Human and mouse cerebellar inhibitory circuits in dystonic crisis and their modulation with therapeutic stimulation

**DOI:** 10.64898/2026.04.01.715774

**Published:** 2026-04-03

**Authors:** Alejandro G. Rey Hipolito, Michael P. Dew, Jason S. Gill, Janelle E. Allen, Karissa A. Chesky, Mariam Hull, Roy V. Sillitoe

**Affiliations:** 1 Department of Pathology & Immunology, Baylor College of Medicine, Houston, Texas, USA; 2 Department of Neuroscience, Baylor College of Medicine, Houston, Texas, USA; 3 Section of Pediatric Neurology and Developmental Neuroscience, Department of Pediatrics Baylor College of Medicine, Houston, Texas, USA; 4 Cerebellum Science Center, Texas Children’s Hospital, Houston, Texas, USA; 5 Jan and Dan Duncan Neurological Research Institute at Texas Children’s Hospital, Houston, Texas, USA

**Keywords:** Cerebellum, dystonia, dystonic crisis, inhibitory cerebellar nuclei, deep brain stimulation, optogenetics

## Abstract

Dystonia is a neurological movement disorder characterized by abnormal muscle contractions that, at their most severe, lead to a life-threatening condition - dystonic crisis. Yet, therapeutic options remain limited by our incomplete understanding of what neural circuits underly the condition. Although cerebellar nuclei neurons are implicated in the origin of baseline dystonic symptoms, it is unclear whether their activity drives dystonic crisis. To explore this role, we found that cerebellar abnormalities and neural inhibition were recurring targets in patients with dystonic crisis, implicating cerebellar inhibitory neurons in its development. We devised a mouse genetics approach to test whether inhibitory cerebellar nuclei neurons (iCNNs) induce dystonic crisis. Directional optogenetic modulation of iCNNs induced dystonic crises on-demand and alleviated spontaneous crises in mice that mimic spontaneous dystonic crises. To investigate whether iCNNs interact with other motor areas during dystonic crisis, we identified monosynaptic iCNN projections to the centrolateral nucleus of the thalamus (CL). Deep brain stimulation of the CL alleviated dystonic crises induced by iCNN photoactivation. Our data uncover a cell type-specific cerebellar origin of dystonic crisis and highlight its therapeutic potential.

## Introduction

Dystonia is a neurological movement disorder characterized by sustained or intermittent muscle contractions that cause abnormal movements, postures, or both (1). In the most severe form of the condition, referred to as dystonic crisis (also termed dystonic storm or status dystonicus), the severity and persistence of dystonia require immediate hospitalization (2). Unfortunately, reaching this stage of devastating symptom intensity leads to mortality in up to 10-12.5% of patients, mostly comprising children with an existing diagnosis of dystonia (3, 4). Relapses are common with a recurrence risk of 25% in the first year, and up to 38.6% at 27 months (5). Treatment options remain limited with variable success and include many complex pharmaceutical strategies or last-resort surgical interventions such as deep brain stimulation (DBS) or an intrathecal or intraventricular baclofen pump (6). Thus, there is an unmet need to develop additional therapeutic options for patients with dystonic crisis, which may be facilitated by identifying the exact neural circuits that drive the condition and targeting them with more precise and effective therapies.

It is unclear what specific neural circuits instigate dystonic crisis and how their dysfunction drives this debilitating condition. However, several lines of evidence suggest that inhibitory neurons and motor structures, especially the cerebellum, contribute to dystonic crisis. Ablation and DBS of motor structures including the globus pallidus and thalamus are therapeutically effective in a subset of patients in dystonic crisis (7), supporting a role for areas within the motor network in this condition. In dystonic crisis, treatments routinely involve drugs that target neural inhibition including benzodiazepines, alpha-2 adrenergic agonists, and anticholinergics(3, 5, 6, 8), suggesting that inhibition in the central nervous system may play a role in the condition just as has been found in other forms of dystonia (9–11). Clinical studies have reported a high incidence of dystonic crisis in patients with cerebral palsy (3, 4, 12, 13), a disorder in which cerebellar dysfunction is thought to play a prominent role (14–16), providing an indirect link to cerebellar dysfunction and dystonic crisis. However, these compelling connections require further investigation.

Although few studies on dystonic crisis exist, preclinical evidence in dystonia highlight a role for cerebellar nuclei neurons, which form the predominant output from the cerebellum to the rest of the motor network (17). In mice, lesioning the superior cerebellar peduncle, the cerebellar outflow tract through which cerebellar nuclei neurons project, was recently shown to cause severe dystonia similar to dystonic crisis (18), suggesting that cerebellar nuclei neurons may contribute to dystonic crisis. In animal studies of paroxysmal dystonia, dystonic episodes correlated with changes in cerebellar nuclei neuron activity. In one study, increased activity of inhibitory cerebellar nuclei neurons (iCNNs) correlated with the onset of dystonic episodes (19), suggesting that the cell type-specificity of cerebellar nuclei neurons may determine their contribution to dystonia. Little is known about the iCNN output pathway, though recent studies have described its termination throughout the brain (20, 21), which may subserve unknown functions in health and disease. Accurately distinguishing the specific roles of the intermingled cerebellar nuclei neurons may be critical for developing better treatments for dystonia and other movement disorders, as was demonstrated for the basal ganglia in a mouse model of Parkinson’s disease (22).

To assess the role of iCNNs in dystonic crisis, we started by examining clinical data to study the possible contribution of the cerebellum and inhibitory neurons to dystonic crisis. To complement this clinical study, we sought to manipulate iCNNs in a mouse model of dystonia, and then test whether motor behaviors that resemble dystonic crisis arise. We therefore devised a conditional mouse genetic model to test the hypothesis that modulation of the iCNN output pathway induces dystonic crisis. Using a previously described animal model of dystonia, we genetically expressed opsins in *Ptf1a*-expressing neurons, including iCNNs, and optogenetically modulated them through their projections in the superior cerebellar peduncle. To examine if and how dystonic crises emerge, we combined this approach with behavioral assays and anatomical examination. We also tested whether iCNNs interact with another motor area implicated in dystonia, the centrolateral nucleus of the thalamus (CL), by using anatomical tracing. We then tested the hypothesis that DBS targeted to the CL would modulate the induced dystonic crises. Our data uncover a cerebellar origin of dystonic crisis and inspire the development of targeted therapies.

## Results

### Cerebellar abnormalities and inhibitory neural function are implicated in the incidence and treatment of dystonic crisis in human pediatric-onset dystonia

**Figure 1:**
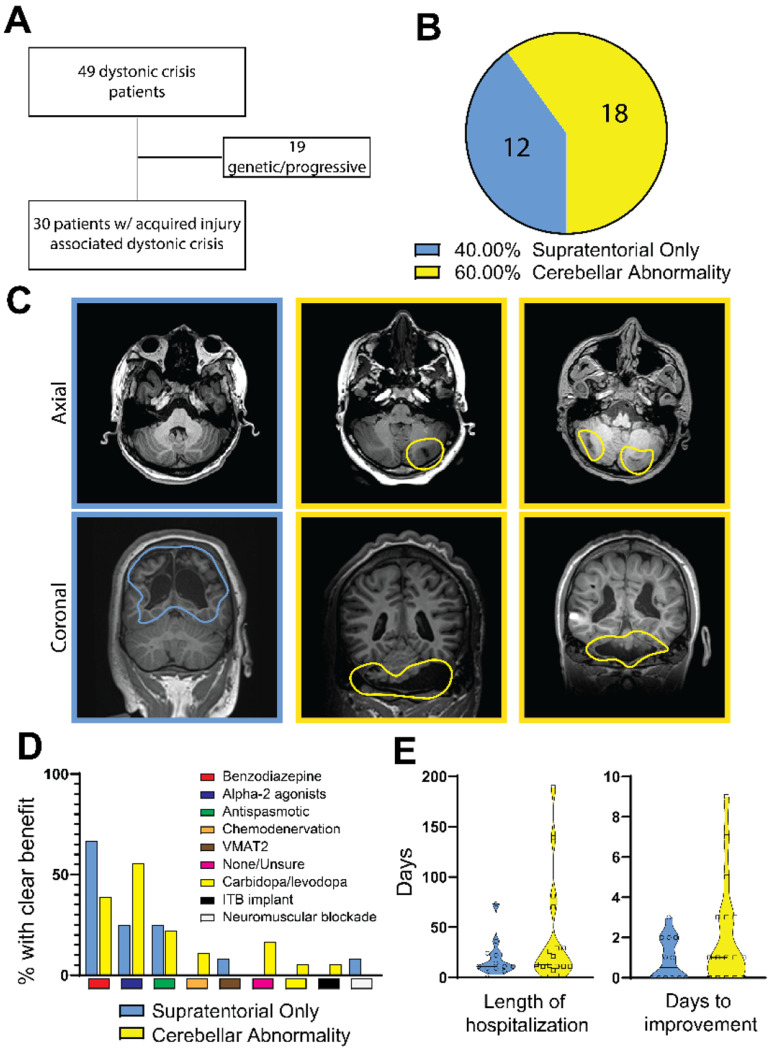
Cerebellar abnormalities and inhibitory neural function are implicated in the incidence and treatment of dystonic crisis. (**A**) Patient flow chart showing the division of 49 patients with dystonic crisis into groups based on the etiology of their dystonia: genetic/progressive (19 patients) or acquired injury (30 patients). (**B**) Percentage of patients admitted for dystonic crisis that had abnormalities in the cerebellum with or without supratentorial findings or only supratentorial abnormalities (blue) on imaging. (**C**) Examples of imaging in patients with supratentorial only injury (left column, outlined in blue) or cerebellar findings (middle and right column, outlined in yellow with abnormalities encircled). The top row depicts the axial plane through the cerebellum on T1 weighted imaging. The top left depicts the cerebellum at the level of the superior cerebellar peduncles, the top middle depicts a sequela of isolated left cerebellar hemispheric stroke, the top right depicts bilateral cerebellar hemispheric encephalomalacia (outlined in blue). The bottom row depicts the coronal plane through the cerebellum and the parietal lobe on T1 weighted imaging. The bottom right shows the normal cerebellum with extensive bilateral periventricular encephalomalacia. The bottom middle and right show patients with extensive bilateral cerebellar encephalomalacia. (**D**) Comparison of therapies that had clear clinical benefit across the 30 patients with acquired injury associated with dystonic crisis. Benzodiazepines and alpha-2 adrenergic agonists were the most widely used and successful. Patients with cerebellar abnormalities responded preferentially to alpha-2 adrenergic agonists rather than benzodiazepines. (**E**) Patients with cerebellar abnormalities tended to have longer lengths of hospitalization (Unpaired t-test with Welch’s correction p=0.167, f=0.001) and days to improvement (Unpaired t-test with Welch’s correction p=0.104, f=0.004) following their admission with dystonic crisis.

As previous clinical data only indirectly linked cerebellar function to dystonic crisis (13, 15), we sought to identify more direct clinical evidence that cerebellar dysfunction leads to dystonic crisis and support further preclinical investigations into the precise cerebellar circuits that may be involved. Accordingly, we investigated the clinical characteristics (Table 1) of patients admitted with dystonic crisis (Supplementary Movie 1) at Texas Children’s Hospital in Houston, Texas between 2015 and 2019. Of the 49 patients admitted, 19 patients were defined as having dystonia associated with a genetic or progressive etiology while the remaining 30 patients (20 males, 10 females; between 3 months and 20 years of age at admission) had an underlying etiology associated with acquired injury (Figure 1A and Table 1). Of the 30 patients with acquired injury, cerebellar abnormalities were observed in the MRI imaging of 18 (60%; 11 males, 7 females; between 3 months and 18 years of age at admission) patients while 12 of 30 patients (40%; 9 males, 3 females; between 3 and 20 years of age at admission) showed imaging with abnormalities exclusively in the supratentorial regions comprising the cerebral hemispheres, basal ganglia, thalamus, and other structures outside the cerebellum (Figure 1B and Table 1), suggesting that cerebellar abnormalities predispose a substantial number of patients to dystonic crisis. The range of cerebellar abnormalities found in patients with dystonic crisis encompassed focal cerebellar only to focal cerebellar with associated supratentorial to diffuse cerebellar injury (Figure 1C). When comparing the therapies that had clear clinical benefit (as determined by parents and clinicians) across the 30 patients with acquired injury, benzodiazepines and alpha-2 adrenergic agonists were the most widely used and successful (Figure 1D). Patients with cerebellar abnormalities responded preferentially to alpha-2 adrenergic agonists rather than benzodiazepines. Interestingly, benzodiazepines and alpha-2 adrenergic agonists have inhibitory effects on the central nervous system (6, 23, 24). In cerebellar nuclei neurons, the corresponding receptors for these two drugs have been identified (25, 26), and alpha-2 adrenergic agonists have been found to modulate their GABA responses (27), indicating a role for GABAergic cerebellar nuclei neurons in the incidence and treatment of dystonic crisis. Patients with cerebellar abnormalities tended to have longer lengths of hospitalization and days to improvement following their admission with dystonic crisis (Figure 1E). To our knowledge, this is the first clinical evidence directly linking cerebellar developmental abnormalities to dystonic crisis. Together, these results strongly support a role for the cerebellum and inhibitory neural circuitry in driving dystonic crisis in human pediatric-onset dystonia. As iCNNs represent the intersection of cerebellar function, neural inhibition, and cerebellar control of the motor network, we hypothesized that iCNNs instigate the mechanisms that trigger dystonic crisis.

### Photoactivation of the iCNN pathway induces dystonic crises reminiscent of the human condition in a mouse model of dystonia, but not in healthy mice

**Figure 2:**
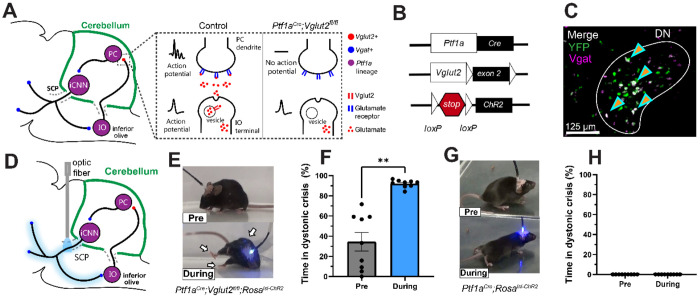
Photoactivation of the inhibitory cerebellar nuclei neuron (iCNN) pathway induces dystonic crises reminiscent of the human condition in *Ptf1a*^*Cre*^*;Vglut2*^*fx/fx*^*;ROSA*^*lsl-Chr2/EYFP*^ mice with existing dystonia, but not in otherwise healthy mice. (**A**) Schematic showing the genetic manipulation in *Ptf1a*^*Cre*^*;Vglut2*^*fx/fx*^ mice that selectively silenced olivocerebellar synapses, which causes dystonia. (**B**) Using the *Ptf1a*^*Cre*^ driver, exon 2 of *Vglut2* was selectively removed and *Vglut2* deleted from the inferior olive. Concurrently, the loxP sites surrounding a stop signal preceding ChR2-YFP were removed, allowing expression of ChR2-YFP in Ptf1a-expressing neurons including inferior olivary neurons, Purkinje cells, and iCNNs. (**C**) *In situ* image showing co-labeling of *YFP* and *Vgat* in the cerebellar nuclei. (**D**) Schematic showing the selective photoactivation iCNNs through their projections in the superior cerebellar peduncle. (**E**) Photoactivation of the iCNN pathway induced a dystonic crisis on-demand. (**F**) In *Ptf1a*^*Cre*^*;Vglut2*^*fx/fx*^*;ROSA*^*lsl-Chr2/EYFP*^ mice, photoactivation of the iCNN pathway significantly increased the percentage of time spent in dystonic crisis when comparing before and during stimulation (n=9 animals; p=0.0039, Wilcoxon matched-pairs signed rank test, two-tailed). (**G**) Photoactivation of the iCNN pathway in healthy *Ptf1a*^*Cre*^*;ROSA*^*lsl-Chr2/EYFP*^ mice resulted in abnormal movements, but not dystonic crises. (**H**) Photoactivation of the iCNN pathway did not influence the percentage of time spent in dystonic crisis when comparing before and during stimulation (n=9 animals). Error bars are defined as standard error of the mean. SCP, superior cerebellar peduncle; PC, Purkinje cell; iCNN, inhibitory cerebellar nuclei neuron; IO, inferior olive; DN, dentate cerebellar nucleus.

Although cerebellar nuclei neurons (19, 28) have been implicated in driving the baseline symptoms of dystonia, it remains unclear whether cerebellar nuclei neurons contribute to dystonic crisis. As previous findings and our clinical results support a role for inhibitory cerebellar neurons, we hypothesized that iCNNs specifically instigate dystonic crisis. As previous studies showed increased activity of iCNNs in an animal model of dystonia during paroxysmal episodes of dystonic symptoms, we reasoned that optogenetic activation of iCNNs could induce the more severe dystonic crisis in an animal model of dystonia. To test this, we devised a mouse genetic model that could simultaneously also be manipulated with optogenetics. Since the development of dystonic crises almost exclusively occur in patients with a pre-existing diagnosis of dystonia(5, 29), we sought to optogenetically manipulate the iCNN pathway in a mouse model with robust dystonic symptoms. The *Ptf1a^Cre^;Vglut2^fx/fx^* mouse (30, 31) was chosen because of the cerebellar specificity of the manipulation that gives rise to dystonia, its *Ptf1a*-specific expression of Cre, and the severity of its dystonic symptoms (Figure 2A). As *Ptf1a* is expressed in iCNNs among other areas (32, 33), it allowed cre-inducible expression within iCNNs to manipulate their activity and visualize their anatomy with genetic precision. In these mutant mice, vesicular glutamate transporter 2 (VGlut2) is genetically excised in *Ptf1a*-expressing neurons. As only inferior olive neurons, a major cerebellar input to cerebellar Purkinje cells, express Ptf1a and Vglut2 (32), this manipulation selectively silences neurotransmission from olivary climbing fibers to the cerebellum (Figure 2A), resulting in a robust dystonic phenotype described previously (30). These mutant mice exhibit dystonic symptoms of mild-to-moderate twisting postures in the limbs, trunk, head, and/or tail interrupted by random and intermittent periods of severe generalized dystonia in several body parts reminiscent of human dystonic crises, making them a great model in which to investigate whether iCNN activity triggers dystonic crisis. Similar to human patients in which the same underlying genetic mutation may result in a spectrum of dystonic symptoms across patients (34, 35), the severity of the underlying dystonic symptoms in the *Ptf1a^Cre^;Vglut2^fx/fx^* mouse can vary between animals. To selectively manipulate the iCNN pathway in these mice using optogenetics, we crossed the *Ptf1a^Cre^;Vglut2^fx/fx^* mice with *ROSA^lsl-Chr2/EYFP^* mice to generate *Ptf1a^Cre^;Vglut2^fx/fx^;ROSA^lsl-Chr2/EYFP^* mice (Figure 2B). To confirm that iCNNs selectively expressed the ChR2 construct in the *Ptf1a^Cre^;Vglut2^fx/fx^;ROSA^lsl-Chr2/EYFP^* mice, we performed *in situ* hybridization assays for the YFP tag in the construct, *Vgat* (inhibitory marker), and *Vglut2* (excitatory marker). Robust co-labeling of YFP and *Vgat*, but not *Vglut2*, was observed in the mutant deletion mice (Figure 2C and Supplemental Figure 1, n=3 animals). To selectively manipulate the iCNN pathway and avoid activation of Purkinje cell terminals (which also express *Ptf1a*) in the cerebellar nuclei, we targeted iCNN axons by bilaterally implanting optical fibers over the superior cerebellar peduncle, the white matter tract through which extracerebellar cerebellar nuclei neuron projections pass (17) (Figure 2D and Supplemental Figure 2). The selective manipulation of cerebellar nuclei neurons by targeting the superior cerebellar peduncle has been similarly employed to create an animal lesion model of dystonic crisis(18) and to therapeutically treat refractory dystonia in patients (36, 37). The behavior of experimental mice in an open field was video recorded and data from two minutes before and during stimulation were analyzed (470 nm light; 50 Hz square pulses; ≥ 1mW at fiber tip). To detect changes in the incidence of dystonic crises, we measured the percentage of time that a mouse spent in a dystonic crisis during each period (before vs during stimulation). Throughout this study, we define a dystonic crisis in mice as observable dystonic limb contractions, limb hyperextensions and/or overt postures in at least two body parts in addition to severely limited ambulation for at least 3 seconds, a sufficient time to confidently determine that ambulation was restricted (rather than due to voluntary resting) and a result of abnormal muscle activity (Supplementary Movie 2).

Photoactivation of the iCNN pathway induced severe dystonic crises on-demand which was clearly observable in videos (Figure 2E and Supplementary Movie 2; i.e.. extended limbs, rigidity throughout the body, stiff tail, splayed digits, toppling over, severely impaired ambulation). The photoinduced dystonic crises were similar to the most severe spontaneous periods of dystonia observed in these mutant mice and reminiscent of the acute, severe episodes of dystonia observed in another murine model of dystonic crisis(18). The photoactivation period was marked by a significant increase in the percentage of time spent in a dystonic crisis during stimulation (92%) compared to before stimulation (34%), supporting that a dystonic crisis was induced (Figure 2F, n=9 animals; p=0.0039, Wilcoxon matched-pairs signed rank test, two-tailed). The photoinduced dystonic crisis occurred within 9 seconds of stimulation onset (Supplemental Figure 3A; n=9 animals), persisted throughout the duration of the light stimulation, and did not end until 38 seconds after the stimulation ended (Supplemental Figure 3B; n=9 animals).

Although dystonic crisis occurs in patients with pre-existing dystonia in most cases, it is possible for otherwise heathy patients to develop a dystonic crisis (5, 29). Likewise, it may be possible that the iCNN pathway may broadly generate general dystonic movements rather than specifically trigger dystonic crisis in the context of a pre-existing dystonic condition. To test this, we repeated this optogenetic experiment in otherwise heathy mice with an opsin (*Ptf1a^Cre^;ROSA^lsl-Chr2/EYFP^*). Photoactivation of the iCNN pathway in healthy mice (*Ptf1a^Cre^;ROSA^lsl-Chr2/EYFP^*) did not result in dystonic crises (Figure 2G and Supplementary Movie 3) as the percentage of time in crisis remained 0 before and during the stimulation (Figure 2H; n=9 animals). However, iCNN photoactivation did lead to abnormal movement and short, intermittent, mild dystonic movements. Almost instantaneously with the onset of stimulation, the mouse was observed to be more tense, lowered its body to the ground as if crawling, widened its hind legs, dragged its trunk along the floor while walking, and appeared to “waddle” with slow and clunky locomotion forward and wide steps (Figure 2G and Supplementary Movie 3). These data suggest that the pre-existing dystonic state of *Ptf1a^Cre^;Vglut2^fx/fx^;ROSA^lsl-Chr2/EYFP^* mice sensitizes the iCNN pathway to trigger dystonic crises. As dystonic crisis predominantly arises in patients with a pre-existing diagnosis of dystonia, these results further support a role for the iCNN pathway in the origin of dystonic crises.

### Photoinhibition of the iCNN pathway alleviates spontaneous dystonic crises in a mouse model of dystonia

**Figure 3:**
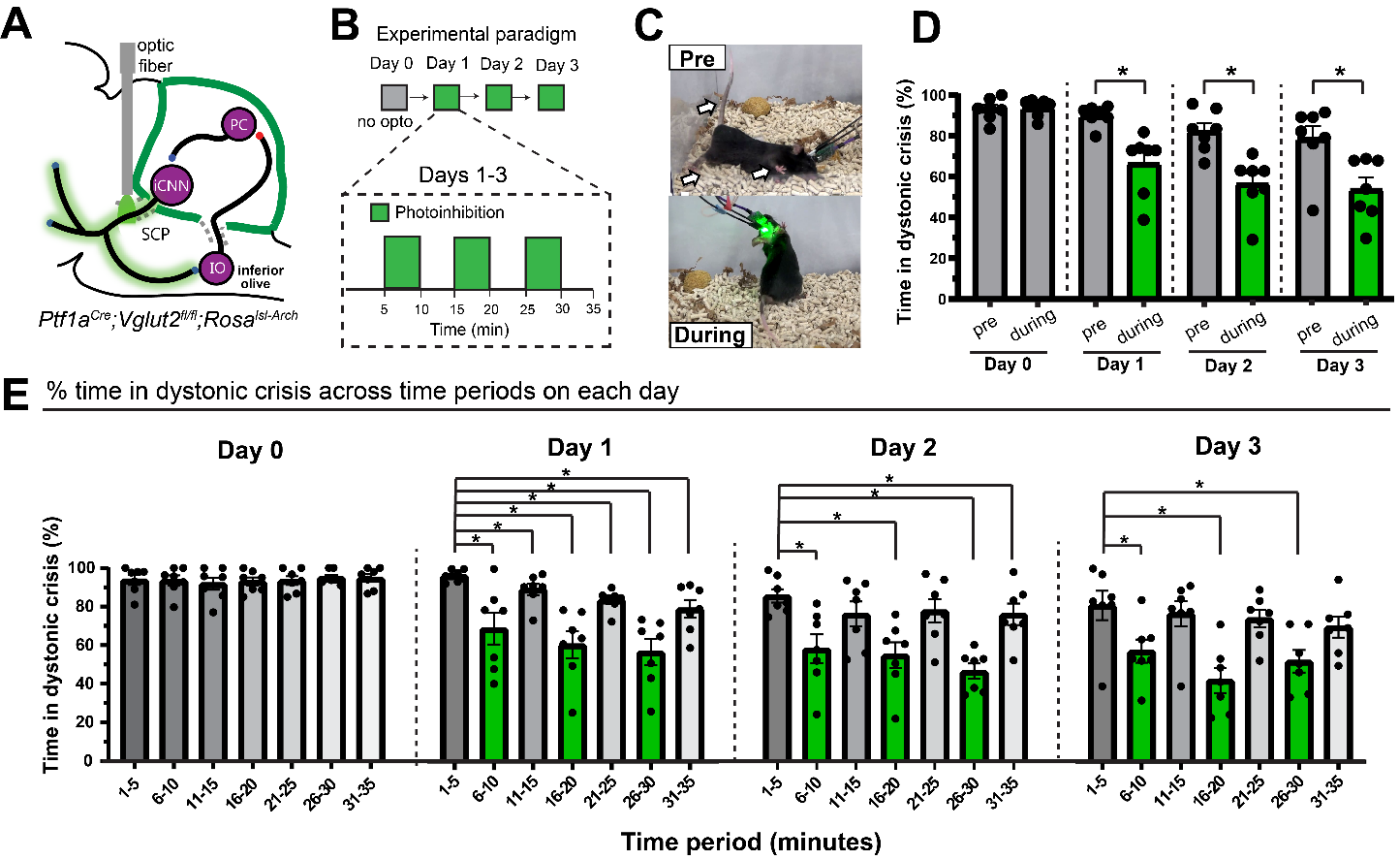
Photoinhibition of the inhibitory cerebellar nuclei neuron (iCNN) pathway alleviates the spontaneous dystonic crises in *Ptf1a*^*Cre*^*;Vglut2*^*fx/fx*^*;ROSA*^*lsl-Arch/EYFP*^ mice with existing dystonia. (**A**) Schematic showing the selective photoinhibition of the iCNN pathway by implanting optical fibers into the superior cerebellar peduncle of *Ptf1a*^*Cre*^*;Vglut2*^*fx/fx*^*;ROSA*^*lsl-Arc*^ mice. (**B**) Schematic showing the four-day experimental paradigm. (**C**) Photoinhibition of the iCNN pathway alleviated dystonic crises in *Ptf1a*^*Cre*^*;Vglut2*^*fx/fx*^*;ROSA*^*lsl-Arch*^ mice as shown in (**D**) by a significant decrease in the percentage of time spent in crisis when comparing pre versus during photostimulation on Day 1 (p=0.0156), Day 2 (p=0.0156), and Day 3 (p=0.0156) (**E**) Photoinhibition on Day 1 reduced dystonic crises across all time periods (for 1-5 vs 6-20, p=0.0312; for 1-5 vs 11-15, p=0.0156; for 1-5 vs 16-20, p=0.0156; for 1-5 vs 21-25, p=0.0156; for 1-5 vs 26-30, p=0.0156; for 1-5 vs 31-35, p=0.0156). Photoinhibition on Day 2 alleviated crises across several time periods (for 1-5 vs 6-20, p=0.0156; for 1-5 vs 11-15, p=0.2345; for 1-5 vs 16-20, p=0.0156; for 1-5 vs 21-25, p=0.2969; for 1-5 vs 26-30, p=0.0156; for 1-5 vs 31-35, p=0.0469). Photoinhibition on Day 3 reduced crises across several time periods (for 1-5 vs 6-20, p=0.0156; for 1-5 vs 11-15, p=0.2188; for 1-5 vs 16-20, p=0.0156; for 1-5 vs 21-25, p=0.1562; for 1-5 vs 26-30, p=0.0156; for 1-5 vs 31-35, p=0.0781). For all statistics: n=7 animals, Wilcoxon matched-pairs signed rank test, two-tailed, error bars are defined as standard error of the mean.

As photoactivation of the iCNN pathway induced dystonic crisis, we reasoned that photoinhibition of the iCNN pathway would alleviate spontaneous dystonic crises in *Ptf1a^Cre^;Vglut2^fx/fx^* mice. To test this, we generated *Ptf1a^Cre^;Vglut2^fx/fx^;ROSA^lsl-Arch^* mice expressing the inhibitory opsin, archaerhodopsin, and targeted photostimulation to the iCNN pathway as previously described (Figure 3A). As we were interested in detecting a reduction in the time spent in dystonic crisis and the severity of dystonia varies across individual mice, we selected *Ptf1a^Cre^;Vglut2^fx/fx^;ROSA^lsl-Arch^* mice that had severe dystonic crises frequently. To address the presence of possible lags in the onset of therapeutic effects of photoinhibition, as previously observed in treatments of dystonic patients (38–41) and animal models (30), we repeated the experiment over four consecutive days. To establish a baseline for how much time the mice spend in dystonic crisis over a 35-minute session without optogenetic manipulation, we performed the experiment without any photostimulation on Day 0 (Figure 3B). To test whether photostimulation influenced the percentage of time spent in dystonic crisis, video was recorded for five minutes before and five minutes during stimulation (561 nm light; continuous light; ≥ 1mW at fiber tip). To increase confidence that any behavioral change was a result of optogenetic manipulation, we repeated the stimulation paradigm three times on each day, resulting in a 35-minute session for each day (Figure 3B). For each day, we averaged the percentage of time spent in crisis before and during the stimulation periods and then compared the resulting values for statistical significance.

To test whether photoinhibition of the iCNN pathway led to a same-day reduction in dystonic crises, we first performed the experiment without any photostimulation on Day 0 (Figure 3b). As expected on Day 0 when no photostimulation was given, we did not find a significant change in the percentage of time spent in dystonic crisis when comparing periods corresponding to before and during stimulation (93% vs 94%; p=0.6875) in severely dystonic *Ptf1a^Cre^;Vglut2^fx/fx^;ROSA^lsl-Arch^* mice (Figure 3D, n=7 animals, Wilcoxon matched-pairs signed rank test, two-tailed). Remarkably, same-day photoinhibition of the iCNN pathway alleviated spontaneous dystonic crises and rescued mobility in these mice (Figure 3C and Supplementary Movie 4) as shown by a significant decrease in the percentage of time spent in dystonic crisis when comparing before versus during photostimulation on Day 1 (90% vs 66%; p=0.0156), Day 2 (82% vs 57%; p=0.0156), and Day 3 (79% vs 54%; p=0.0156)(Figure 3D; n=7 animals, Wilcoxon matched-pairs signed rank test, two-tailed).

As our previous analysis for each day averaged the effects of three stimulations over 35 minutes, we wondered whether the observed therapeutic effects were triggered immediately by the first stimulation or whether they developed progressively over the course of the three stimulations of each day. To test this, the percentage of time in dystonic crisis across five-minute periods on a given day was compared (Figure 3E). On Day 0, when comparing the first five minute period (1–5) to each subsequent five-minute time period (6–10, 11–15, 16–20, 21–25, 26–30, 31–35), there was no significant change (Figure 3E, n=7 animals; for 1-5 vs 6-20, p=0.7500; for 1-5 vs 11-15, p=0.0625; for 1-5 vs 16-20, p=0.9219; for 1-5 vs 21-25, p=0.8750; for 1-5 vs 26-30, p=0.6719; for 1-5 vs 31-35, p=0.8438; Wilcoxon matched-pairs signed rank test, two tailed). When making the same comparison on Day 1, we found a significant reduction in dystonic crises across all time periods (Figure 3E, n=7 animals; for 15 vs 6-20, p=0.0312; for 1-5 vs 11-15, p=0.0156; for 1-5 vs 16-20, p=0.0156; for 1-5 vs 21-25, p=0.0156; for 1-5 vs 26-30, p=0.0156; for 1-5 vs 31-35, p=0.0156; Wilcoxon matched-pairs signed rank test, two tailed). On Day 2, we found a significant reduction in dystonic crisis across several time periods (Figure 3E, n=7 animals; for 1-5 vs 6-20, p=0.0156; for 1-5 vs 11-15, p=0.2345; for 1-5 vs 16-20, p=0.0156; for 1-5 vs 21-25, p=0.2969; for 1-5 vs 26-30, p=0.0156; for 1-5 vs 31-35, p=0.0469; Wilcoxon matched-pairs signed rank test, two tailed). Day 3 showed a similar significant reduction during stimulation periods (Figure 3E, n=7 animals; for 1-5 vs 6-20, p=0.0156; for 1-5 vs 11-15, p=0.2188; for 1-5 vs 16-20, p=0.0156; for 1-5 vs 21-25, p=0.1562; for 1-5 vs 26-30, p=0.0156; for 1-5 vs 31-35, p=0.0781; Wilcoxon matched-pairs signed rank test, two tailed). As the significant reductions in the time spent in dystonic crisis on Day 2 and Day 3 predominantly occur during stimulation periods and not non-stimulation periods, they support the idea that the alleviation of dystonic crises is a consequence of our optogenetic manipulation rather than other confounding variables like exhaustion or familiarity with the environment. These results further suggest that photoinhibition of the iCNN pathway has short-term, same-day, cumulative therapeutic effects on dystonic crisis.

To explore whether any long-term, multiday, cumulative therapeutic effects may have occurred across days, the percentage of time in dystonic crisis was compared across the pre stimulation periods and during stimulation periods across days (Supplemental Figure 4). For the pre stimulation periods when comparing Day 1 (the first day of stimulation) against Day 2 and Day 3, we report a trend toward a reduction in dystonic crises, but it is not statistically significant (Supplemental Figure 4A, n=7 animals; for Day 1 vs Day 2, n=0.0625; for Day 1 vs Day 3, n=0.0781; Wilcoxon matched-pairs signed rank test, two tailed). For the during stimulation periods, we found a significant reduction in dystonic crises when comparing Day 1 to Day 2 and Day 3 (Supplemental Figure 4B, n=7 animals; for Day 1 vs Day 2, n=0.0312; for Day 1 vs Day 3, n=0.0156; Wilcoxon matched-pairs signed rank test, two tailed). These results suggest that the photoinhibition of the iCNN pathway also has long-term, multiday, cumulative therapeutic effects on dystonic crisis.

Together, these results suggest that photoinhibition of iCNN activity immediately alleviates spontaneous dystonic crises in mice with severe dystonia, and that these beneficial therapeutic effects accumulate over time.

### iCNNs send direct projections to the centrolateral nucleus of the thalamus (CL)

**Figure 4:**
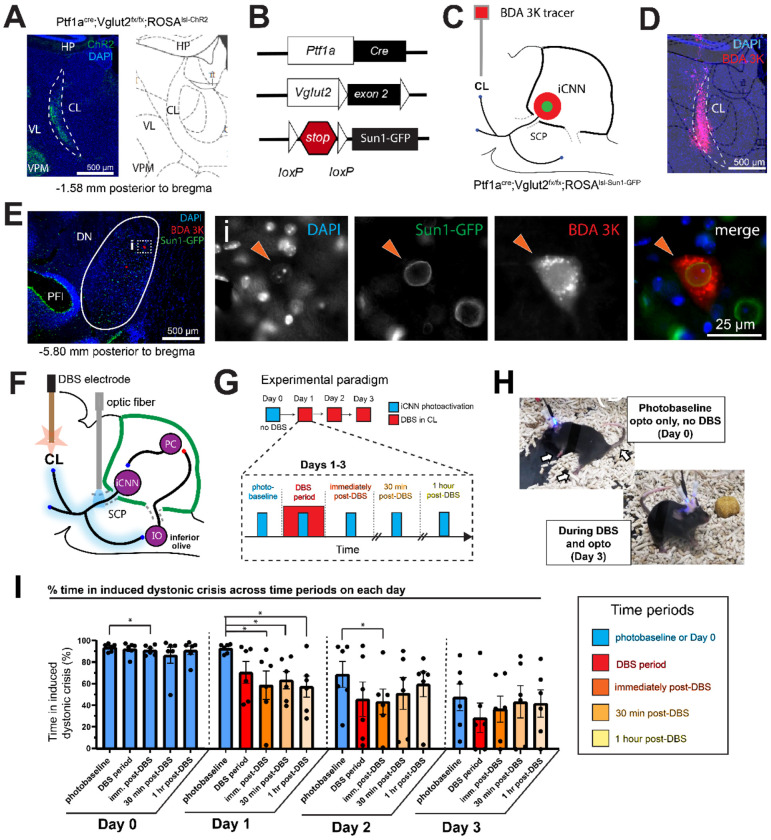
Inhibitory cerebellar nuclei neurons (iCNNs) interact with the centrolateral nucleus of the thalamus (CL) during dystonic crisis. (**A**) Immunohistochemical staining in *Ptf1a*^*Cre*^*;Vglut2*^*fx/fx*^*;ROSA*^*lsl-Chr2/EYFP*^ mice revealed ChR2-YFP-labeled projections in the CL. (**B**) Schematic of genetics in *Ptf1a*^*Cre*^*;Vglut2*^*fx/fx*^*;ROSA*^*lsl-Sun1-GFP*^ mice that allowed localized GFP expression in the somas of Ptf1a-expressing neurons. (**C**) Experimental setup to identify iCNNs projecting to the CL. Retrograde BDA 3K tracer was injected into CL of *Ptf1a*^*Cre*^*;Vglut2*^*fx/fx*^*;ROSA*^*lsl-Sun1-GFP*^ mice as in (**D**), and iCNNs were co-labeled with BDA 3K and GFP as shown in (**E**, inset **i**) (n=4 mice). (**F**) Schematic showing the selective photoactivation of iCNN projections in the SCP and DBS of the CL. (**G**) Four-day experimental setup showing the optogenetic and DBS paradigms during the five distinct time periods for each day. **(H**) Representative images showing how DBS alleviated dystonic crisis induced by photoactivation of the iCNN pathway. (**I**) Dystonic crises induced by photoactivation of the iCNN pathway did not change greatly across time periods on Day 0 though a statistical difference was detected when comparing the photobaseline to immediately post-DBS (p=0.0312). Induced crises were substantially reduced on Day 1 when comparing the photobaseline to immediately post-DBS (p=0.0312), 30 minutes post-DBS (p=0.0312), and 1 hour post-DBS (p=0.0312) and on Day 2 when comparing the photobaseline to immediately post-DBS (p=0.0312). For all statistics: n=6 animals, Wilcoxon matched-pairs signed rank test, two-tailed, error bars are defined as standard error of the mean. CL, centrolateral nucleus of the thalamus; HP, hippocampus; VL, ventrolateral thalamus; VPM, ventroposterior thalamus; Pfl, paraflocculus; DN, dentate cerebellar nucleus. SCP, superior cerebellar peduncle; DBS, deep brain stimulation; iCNN, inhibitory cerebellar nuclei neuron.

As dystonia is thought to result from aberrant interactions across structures in the motor network (42), we reasoned that the iCNN pathway interacts with other structures in the motor network to control the onset of a dystonic crisis. Emerging evidence in patients (34, 43–45) and animal models (30, 46, 47) support a role for the cerebello-thalamo-basal ganglia pathway in several forms of dystonia, and anatomical studies in non-human primates and animal models have revealed that the cerebellar nuclei interact with the basal ganglia through the CL (48, 49). However, it is thought that these cerebello-thalamic projections predominantly originate from excitatory, not inhibitory, cerebellar nuclei neurons (20, 49, 50). Moreover, it remains unknown whether the cerebello-thalamo-basal ganglia pathway contributes to dystonic crisis. To investigate whether iCNNs form a part of the cerebello-thalamo-basal ganglia pathway, we hypothesized that there may be a previously unknown monosynaptic projection from iCNNs to the CL. In *Ptf1a^Cre^;Vglut2^fx/fx^;ROSA^lsl-Chr2/EYFP^* mice, we identified axons labeled with ChR2-EYFP in the CL (Figure 4A), suggesting that there may indeed be monosynaptic iCNN projections. However, as these projections could originate from any *Ptf1a*-expressing region (including the hypothalamus, retina, spinal cord, cerebellum, brainstem, or forebrain), we designed an experiment to determine whether iCNNs have a robust monosynaptic projection to the CL using a combination of intersectional genetics and injected neural tracers. To genetically label only the cell bodies of *Ptf1a*-expressing neurons like iCNNs, we crossed *Ptf1a^Cre^;Vglut2^fx/fx^* with a cre-dependent Sun1 (a small nuclear envelope protein) reporter line to generate *Ptf1a^Cre^;Vglut2^fx/fx^;ROSA^lsl-Sun1-GFP^* mice (Figure 4B). To retrogradely label the cell bodies of iCNNs that may project to the CL, we bilaterally injected BDA 3K (a predominantly retrograde neural tracer (51, 52)) into the CL (Figure 4C–D). We found co-labeling of DAPI, BDA 3K, and Sun1-GFP in the cerebellar nuclei (Figure 4E, **inset i; orange ticks**), suggesting that iCNNs monosynaptically project to the CL. We found co-labeling of these three markers across all three major cerebellar nuclei comprising the fastigial, interposed, and dentate cerebellar nuclei (Supplemental Figure 5). These data identify a specific cerebello-thalamic anatomical pathway through which iCNNs could control and/or contribute to the onset of dystonic crisis.

### DBS of the CL alleviates dystonic crises induced by iCNN photoactivation

As our data showed that iCNN photoactivation induces dystonic crises and that iCNNs project to the CL, we sought to test whether iCNNs and the CL interact to control the onset of dystonic crisis and reflect a possible therapeutic node. Specifically, we wondered whether CL activity is required to induce the dystonic crises triggered by the iCNN pathway. This importance of CL activity in dystonia has been highlighted by previous studies showing that the disruption of CL activity with pharmacological agents, electrolytic lesions, or DBS can alleviate dystonic symptoms in animal models of dystonia (18, 30, 46, 49) including the *Ptf1a^Cre^;Vglut2^fx/fx^* model. Thus, we reasoned that if the iCNN pathway, specifically, induces dystonic crises through the CL, then DBS of the CL will alleviate the dystonic crises induced by iCNN photoactivation.

To test this in the *Ptf1a^Cre^;Vglut2^fx/fx^;ROSA^lsl-Chr2/EYFP^* mice, we unilaterally implanted an optical fiber over the superior cerebellar peduncle and bilaterally implanted bipolar DBS electrodes into the CL (Figure 4F and Supplemental Figure 6). To account for possible delays in the therapeutic effect of DBS as previously observed in patients and animal models, we repeated the experiment over four consecutive days. Each day consisted of an 86-minute session divided into five distinct periods: photobaseline, DBS period, immediately post-DBS, 30 minutes post-DBS, and 1 hour post-DBS (Figure 4G). During each distinct period, a 2-minute stimulation of blue light (470 nm light; 50 Hz square pulses; ≥ 1mW at fiber tip) was administered followed by 2 minutes of no light stimulation. This paradigm was repeated three times for each distinct period. The percentage of time spent in a dystonic crisis was quantified for each of the three light pulses, and the average of these values was analyzed. To establish a baseline for how much time the mice spend in dystonic crises induced by iCNN photoactivation, we performed this experiment without any DBS on Day 0 (Figure 4G). We administered DBS on Days 1-3. We only performed DBS in the DBS period for 10 minutes (130 Hz, 60us, 30uA), starting with the onset of the first light pulse and ending with the offset of the third light pulse.

First, we analyzed whether CL-DBS led to same-day changes in the time in induced dystonic crisis across the five time periods (Figure 4I). On Day 0, the percentage of time in induced crisis across the five time periods remained mostly the same (between 83–94%), though there was a statistically significant reduction during photobaseline compared to the immediately post-DBS period (94% vs 91%;p=0.0312) (Figure 4I; n=6 mice, Wilcoxon matched-pairs signed rank test, two tails). This suggested that iCNN photoactivation reliably and consistently induced dystonic crises throughout the 86-minute session. On Day 1, when comparing between the photobaseline and all subsequent time periods after DBS was administered, we found a marked reduction in the time spent in induced crisis when comparing the same-day photobaseline to during DBS (93% vs 70%; p=0.0625), immediately post-DBS (93% vs 58%; p=0.0312), 30 minutes post-DBS (93% vs 63%; p=0.0312), and 1 hour post-DBS (93% vs 57%; p=0.0312)(Supplementary Movie 5 and Figure 4I, n=6 animals, Wilcoxon matched-pairs signed rank test, two tails). This progression suggests a short-term, delayed therapeutic effect of CL-DBS on iCNN-mediated dystonic crises. On Day 2, while the same general trend in reduced time in induced dystonic crisis following CL-DBS was observed, there was only a statistically significant reduction from the same-day baseline to immediately post-DBS (69% vs 44%; p=0.0312)(Figure 4I, n=6 animals, Wilcoxon matched-pairs signed rank test, two tails). On Day 3, we did not find statistically significant changes though the same general trend in reduced crisis time following DBS can be seen (Figure 4I). Importantly, a trending decrease in dystonic crises across days can be appreciated (Figure 4H–I), especially in the photobaseline periods, which may explain the lack of statistically significant comparisons on Days 2-3. These data suggest that CL-DBS causes long-term, multi-day, therapeutic changes that alleviate dystonic crises induced by iCNN photoactivation.

To further explore whether CL-DBS led to long-term, multi-day therapeutic changes in the induced dystonic crisis time, we compared the time in induced dystonic crisis for each period across Days 0-3 (Supplemental Figure 7). When comparing the photobaseline period across days (Supplemental Figure 7A), we observed a clear reduction in the time in induced crisis on Day 2 (69%) and Day 3 (48%) compared to Day 0 (94%) and Day 1 (93%). This corresponded to the days after CL-DBS had been administered at least once, suggesting that CL-DBS likely led to this long-term therapeutic change in dystonic crisis induction. However, we only detected a statistically significant reduction when comparing Day 0 with Day 3 (Supplemental Figure 7A, n=6 animals, p=0.0312, Wilcoxon matched pairs signed rank test, two tails). For the DBS period, when comparing against the time in crisis on Day 0 (92%), we observed a trending but non-significant reduction on Day 1 (92% vs 70%; p=0.1562) and a statistically significant reduction on Day 2 (92% vs 46%; p=0.0312) and Day 3 (92% vs 29%; p=0.0312)(Supplementary Movie 5 and Supplemental Figure 7A, n=6 animals, Wilcoxon matched-pairs signed rank test, two tails). For immediately post-DBS, the induced attack time on Day 0 significantly decreased on Day 1 (91% vs 58%; p=0.0312), Day 2 (91% vs 44%; p=0.0312), and Day 3 (91% vs 37%; p=0.0312)(Supplemental Figure 7A, n=6 animals, Wilcoxon matched-pairs signed rank test, two tails). For 30 minutes post-DBS, the induced crisis time on Day 0 decreased on Day 1 (86% vs 63%; p=0.0312), Day 2 (86% vs 51%; p=0.01562), and Day 3 (86% vs 44%; p=0.0312), though it only reached statistical significance for Days 1 and 3 (Supplemental Figure 7A, n=6 animals, Wilcoxon matched-pairs signed rank test, two tails). For 1 hour post-DBS, the induced crisis time on Day 0 decreased significantly on Day 1 (92% vs 61%; p-0.0156), Day 2 (92% vs 64%; p-0.0156), and Day 3 (93% vs 49%; p=0.0156)(Supplemental Figure 7A, n=6 animals, Wilcoxon matched-pairs signed rank test, two tails). Together, these results suggest that the iCNN pathway and CL interact to control the onset of dystonic crises, and that CL-DBS may provide prolonged and cumulative therapeutic effects for alleviating dystonic crises.

## Discussion

Little is known about iCNNs, and their functional contribution has been historically limited to motor learning and coordination (53–57). However, recent studies have increasingly supported a more prominent role for this subtype of cerebellar nuclei neurons, showing that they have distinct anatomical, electrophysiological, and protein expression properties (20, 21, 53) and that they may be involved in non-motor processes (58). For example, Judd et al. 2021 found that iCNNs restricted to the anterior interposed cerebellar nucleus send non-canonical, widespread projections to motor and non-motor areas (20). Our data suggest that iCNNs control the onset of dystonic crisis through their interaction with the CL. To our knowledge, this is the first evidence to support a role for iCNNs in disease, to describe a functional role for its extracerebellar projections outside the inferior olive, and to characterize its interaction with the motor network in disease. This finding of a precise, cell type-specific circuit may hold promise as a therapeutic avenue for intervention in patients with dystonic crisis or other refractory and debilitating forms of dystonia, with our data raising opportunities for further preclinical and clinical investigation. The significance of uncovering the distinct functions of intermingled neurons, like those of the cerebellar nuclei, has been highlighted in other brain areas and diseases. In a mouse model of Parkinson’s disease, Spix et al. 2021 leveraged differences in cell-type specific neurons within the external globus pallidus to develop an optimized protocol for DBS, leading to longer-lasting therapeutic effects when compared to conventional DBS (22). Adapting this approach for optimizing DBS directed to iCNNs and its projections in the CL may yield similarly promising therapeutic results for patients with dystonia or other cerebellar-related disorders. Whether iCNNs contribute to other diseases remains an open question, although we anticipate that our results will encourage the further exploration of this possibility and the therapeutic utility of this understudied neuronal population.

We found that DBS of the CL alleviated dystonic crises induced by iCNN photoactivation. While this result suggests that both structures interact to control the onset of dystonic crises, it does not reveal the exact nature of how this interaction occurs. Future studies are needed to investigate whether this therapeutic effect relies solely on the iCNN-CL projection, how iCNN activity modulates CL activity on a moment-to-moment basis during crisis induction, and how CL activity may further influence other connected motor structures beyond the basal ganglia. Elucidating these features may pinpoint the complete repertoire of neural substrates in the motor network that collaborate to induce dystonic crises and how they interact to drive the condition. Answering these questions may reveal critical disease-modifying therapeutic strategies.

Our study utilizes a unique intersectional genetic approach leveraging the developmental, *Ptf1a*-dependent expression of cre, which allowed access to iCNNs in all cerebellar nuclei. This may explain, at least in part, why our anatomical results may appear inconsistent with a recent study. Unlike our study, Judd et al. 2021 did not find that iCNNs project to the CL, which may be due to their viral injection strategy of cre-dependent fluorescent proteins in Vgat-cre mice (20). Specifically, the potential iCNNs that could be labeled with this technique were restricted primarily to the injected region (the anterior interposed cerebellar nucleus, a portion of one of three cerebellar nuclei) and dependent on the variability of targeting and viral expression, which may have not included those iCNNs that do project to the CL. In fact, we found CL-projecting iCNNs in all three cerebellar nuclei (Supplemental Figure 5).

Our approach of leveraging the *Ptf1a*-specific expression of opsins in iCNNs and the anatomical restriction of their projections in the superior cerebellar peduncle provided an opportunity to selectively modulate their activity while avoiding unwanted photomodulation of nearby regions. While the robust ChR2-YFP expression in the targeted region of the superior cerebellar peduncle validated our approach, we cannot exclude the possibility that other observable nearby Ptf1a-expressing axons in the Kolliker-Fuse nucleus (KF), supratrigeminal nucleus (Su5), and motor trigeminal nucleus (Mo5) may have contributed to the optogenetically induced behaviors we observed (Supplemental Figure 2A’). Similarly, *Ptf1a*-expressing projections from Purkinje cells in the parabrachial nucleus, which predominantly target areas around the superior cerebellar peduncle around 5.6 mm posterior to bregma, may have been photomodulated from our approach (59). Nonetheless, this is unlikely as the light spread from our setup was calculated to not sufficiently illuminate these regions for photomodulation (see Methods – optical fiber implantation and stimulation); moreover, these regions are known to be involved in breathing, the sensorimotor control of the trigeminal nerve, and place preference, which would be unlikely drivers of dystonic crisis.

Although our experiments support a role for the iCNN pathway in controlling the onset of dystonic crisis, it is highly likely that excitatory cerebellar nuclei neurons also contribute to the condition as supported by studies into forms of paroxysmal dystonia. Wu et al. 2025 showed that selective photoactivation of excitatory cerebellar nuclei neurons induced dystonia-like movements in healthy mice, and that selective ablation of these neurons alleviated attacks in mouse models of paroxysmal dystonia (19). Other studies similarly supported a contribution for excitatory cerebellar nuclei in paroxysmal dystonia though their optogenetic methods technically did not exclude iCNNs from being manipulated in their studies. While iCNNs project outside the cerebellum and to the cerebellar cortex, they are also known to collateralize widely and form local synaptic connections with excitatory cerebellar nuclei neurons (17, 20, 53); thus, inhibitory and excitatory cerebellar nuclei neuron activity are tightly linked. Furthermore, Judd et al. 2021 showed that the excitatory and inhibitory cerebellar nuclei share many inputs and extracerebellar outputs (20), suggesting that they work synergistically. One possibility is that the excitatory neurons play a major role in the severity of baseline dystonia whereas the inhibitory neurons set the threshold for the dystonic crisis. While we cannot exclude the possibility that our optogenetic manipulation of iCNN activity through ChR2 could have caused backpropagation and influenced excitatory cerebellar nuclei neurons, it still does not discount our finding that direct manipulation of iCNN activity ultimately contributed to the phenotypes we observed in this study, regardless of what other structures may have been involved or required. Future studies are needed to investigate how the excitatory and cerebellar nuclei neurons interact to control dystonic crisis and other forms of dystonia, which may reveal population-specific strategies for designing therapeutic interventions to address the broader symptomology that collectively impact patients with dystonia.

## Materials and Methods

### Clinical methods

We performed a retrospective chart review of patients admitted to Texas Children’s Hospital for the primary diagnosis of status dystonicus (also termed dystonic crisis) using (ICD9/10 codes) from 2015–2020 under approved IRB (H-48706). Demographics, medical history, and clinical notes were reviewed for all patients and information included etiology, laboratory studies, imaging, treatments, dosing, response, length of stay, and recurrence of symptoms. Efficacy of treatment was determined by patient/physician report as documented within the clinical notes.

### Animals

All experiments were performed following the relevant ethical regulations for animal testing. All mice were housed in an AALAS-accredited facility on a 14h/10h light/dark cycle. Mice of both sexes were studied. Husbandry, housing, euthanasia, and experimental guidelines were reviewed and approved by the Institutional Animal Care and Use Committee (IACUC) of Baylor College of Medicine (protocol number: AN-5996).

Dr Chris Wright (Vanderbilt University School of Medicine) kindly provided the *Ptf1a^Cre^* mice(30, 33). We purchased the *Vglut2^floxed^* (*Vglut2^fx^*, #012898) mice (60) from The Jackson Laboratory (Bar Harbor, ME, USA) that contained *LoxP* sites surrounding exon 2 of the gene encoding vesicular glutamate transporter 2 (VGLUT2). These two mouse lines were bred out as described previously to generate dystonic *Ptf1a^Cre^;Vglut2^fx/fx^* mice (30). To develop dystonic transgenic mice with restricted Sun1 expression, we crossed these *Ptf1a^Cre^;Vglut2^fx/fx^* mice to heterozygous or homozygous *ROSA^lox-stop-lox-Sun1^*λ lines (*B6;129-Gt(ROSA)26Sor^tm5(CAG-Sun1/sfGFP)Nat^/J*, Strain #:021039, Jackson Laboratory, Bar Harbor, ME, USA). To gain light-sensitive control over inhibitory cerebellar nuclei neurons with channelrhodopsin (ChR2) or archaerhodopsin (Arch), we crossed *Ptf1a^Cre^;Vglut2^fx/fx^* or *Ptf1a^Cre^* males to either *Ai32 ROSA^lox-stop-lox-ChR2-EYFP^* (B6;129-Gt(ROSA)26Sor^tm32(CAG-COP4*H134R/EYFP)Hze^/J, Strain #:024109, Jackson Laboratory, Bar Harbor, ME) or *Ai40D* (*Gt(ROSA)*26Sor^tm40.1(CAG-aop3/GFP)Hze/J^, Strain #:021188, Jackson Laboratory, Bar Harbor, ME) females(61). These genotypes of all offspring were confirmed by PCR of digested tissue (tail snips or ear punches) and confirmed by presence of Sun1-GFP, ChR2-EYFP, or Arch-EYFP signal in select neurons (it is important to ensure that cre-dependent expression is not present in all neurons, which would indicate germline transmission). For experimental studies, the day of birth was considered as postnatal day 0 (P0).

### Surgery

Surgery for awake optogenetics was performed as described previously (62). In brief, mice were anesthetized with 4% isoflurane in an induction chamber until unresponsive to the toe-pinch reflex. After transferring to a stereotaxic platform (David Kopf Instruments, Tujenga, CA, USA), isoflurane was lowered and maintained at 2% for the duration of surgery. Using aseptic technique and a sterile work field, the mouse was cleaned and prepped and the skin above the skull opened, and a small craniotomy of approximately 1 mm in diameter made above the target region(s). Following the implantation of optical fibers or deep brain stimulation electrodes as described below, all animals were allowed to recover for at least three days to a maximum of one week after surgery. The following surgical techniques were either employed as individual experiments or combined depending on the requirements of the experiment.

### Optical fiber implantation and stimulation

Optical fiber implant surgeries began as described above. One (for unilateral) or two (for bilateral) small craniotomies of about 1mm were performed dorsal to the target region through which optical fibers (Thorlabs, Newton, NJ, USA; #FT200UMT) were lowered into the target region: the superior cerebellar peduncle (5.02 mm posterior and ±1.25 mm lateral to Bregma, 2.5 mm ventral from the brain surface). Optical fibers had been previously glued into ceramic ferrules (Thorlabs, Newton, NJ, USA; #CFLC230-10), polished (Thorlabs, Newton, NJ, USA; #LF5P, #LF3P, #LF1P, #LF03P, #LFCF), epoxied to each other at a set distance using Bondic, (a UV light-activated bonding agent; Bondic, Niagara Falls, NY, USA), and placed inside ceramic mating sleeves (Thorlabs, Newton, NJ, USA; #ADAL 1–5) prior to implantation. The optical fibers and 1-2 metal rods, used for holding the mouse’s head stable while connecting the optical fiber patch cables, were affixed to the skull with C&B Metabond (Parkell, Inc., Edgewood, NY, USA, SKU: S380) and Teets ‘Cold Cure’ Dental Cement (A-M Systems, LLC, Carlsborg, WA, USA, Catalog #525000 and #526000).

Stimulation patterns were programmed and recorded using Spike2 software and delivered using a CED Power1401 data acquisition interface (CED, Cambridge, UK) to control a 470 nm LED (Thorlabs, Newton, NJ, USA; #M470F4) or a 561 nm laser (CrystaLaser, Reno, NV, USA; # CL561-050). Maximum LED power at the end of the implanted fiber was measured to be ~3.0 mW and stimulation consisted of 50 Hz square pulses of blue 470 nm light. Maximum laser power at the end of the implanted fiber was measured to be ~10 mW and stimulation consisted of a continuous pulse of green 561 nm light. The light powers were calculated to be capable of driving neurons at a distance of about 0.2 mm away or 0.4 mm away from the fiber tip for 470 nm LED light and 561 nm laser light, respectively, using the threshold of 1 mW/mm^2^ (63).

### Tracer injections

This procedure for tracer injections has been described in detail previously (64). Briefly, prior to surgery, the glass micropipettes (Borosilicate Standard Wall without Filament glass capillaries, 1.5 mm o.d. × 0.86 mm i.d. × 75 mm length) were prepared using a micropipette puller with the heat level set at 60 on the Step 1 setting of the Narishige PC-10 Puller and with two heavy weights and one light weight attached. Prior to use, the micropipette tips were clipped using forceps to ensure dispensability of neural tracer, the micropipette was attached to the Nanoject II, and ~1.0 microliters of BDA 3k were loaded into the micropipette. Then, the surgery proceeded as described above for the optical fiber implantation. Except, instead of optical fibers, the filled micropipette was lowered slowly into the stereotaxic coordinates for the centrolateral nucleus of the thalamus (1.50 mm posterior and ±0.80 mm lateral to Bregma, 3.25 mm ventral from the brain surface). The Nanoject II was set to inject the smallest possible amount at the slowest available speed, and injections were given at 10 second intervals until ~75 nL of BDA 3K were injected. After waiting five minutes, the micropipette was slowly taken out of the brain. The procedure was repeated for the contralateral site. After injections, the craniotomies were covered with antibiotic and the skull and skin were sealed with C&B Metabond (Parkell, Inc., Edgewood, NY, USA, SKU: S380) and Teets ‘Cold Cure’ Dental Cement (A-M Systems, LLC, Carlsborg, WA, USA, Catalog #525000 and #526000). Mice were transcardially perfused 9-12 days after the tracer injection procedure to allow the BDA 3K to penetrate neurons and travel retrogradely into the soma of input neurons (51, 52).

### DBS electrode implantation and stimulation

DBS electrode implant surgeries were performed exactly as the optical fiber implantation surgeries. Except, instead of optical fibers, custom 50 mm twisted bipolar Tungsten DBS electrodes were used (PlasticsOne, Roanoke, VA, USA; #8IMS303T3B01) and targeted to the centrolateral nucleus of the thalamus (1.50 mm posterior and ±0.78 mm lateral to Bregma, 2.55 mm ventral from the brain surface). For bilateral implantation, the two electrodes were fixed together using Bondic, a UV light-activated bonding agent (Bondic, Niagara Falls, NY, USA). The electrodes and 1-2 metal rods, used for holding the mouse’s head stable while connecting the DBS cables, were secured to the head with C&B Metabond (Parkell, Inc., Edgewood, NY, USA, SKU: S380) and Teets ‘Cold Cure’ Dental Cement (A-M Systems, LLC, Carlsborg, WA, USA, Catalog #525000 and #526000).

For DBS of the centrolateral nucleus of the thalamus, we used a Multichannel Systems Pulse generator (AMPI, Jerusalem, Israel) programmed to deliver 30 μA, 60 μs biphasic square pulses at 130 Hz.

### Behavioral analyses

The *Ptf1a^Cre^;Vglut2^fx/fx^* mutant mice exhibit dystonic symptoms of mild-to-moderate twisting postures in the limbs, trunk, head, and/or tail interrupted by random and intermittent attacks of generalized dystonia in several body parts reminiscent of dystonic crises (30). Similar to human patients in which the same underlying genetic mutation may result in a spectrum of dystonic symptoms across patients (34, 35), the severity of the underlying dystonic symptoms in the *Ptf1a^Cre^;Vglut2^fx/fx^* mouse can vary between animals. Spontaneous and optogenetically-induced dystonic crises were marked by prolonged and severe dystonic posturing including extended limbs, rigidity throughout the body, stiff tail, splayed digits, toppling over, and severely impaired ambulation for at least three seconds. Optogenetically-induced attacks persisted throughout the length of the optogenetic stimulation, except during or following DBS of the centrolateral nucleus of the thalamus.

The behavior of experimental mice in an open field was video recorded and video data from before and during stimulation were analyzed. For experiments involving photoinhibition (Figure 3B) and DBS of the CL (Figure 4G), three trials were given each day for four consecutive days. To detect potential changes in the incidence of dystonic crises, we measured the percentage of time that a mouse spent in a dystonic crisis during each period (before vs during stimulation), calculated the average for each period on a given day if applicable, and made statistical comparisons using the Wilcoxon matched-pairs signed rank test. Throughout this study, we define a dystonic crisis as observable dystonic muscle contractions or postures in at least two body parts in addition to severely limited ambulation for at least 3 seconds, a sufficient time to confidently determine that ambulation was limited (rather than due to resting) and a result of abnormal muscle activity.

For bilateral photoactivation of the superior cerebellar peduncle in *Ptf1a^Cre^;Vglut2^fx/fx^;ROSA^lsl-Chr2/EYFP^* mice, two-minute periods were video recorded before and during stimulation. For bilateral photoinhibition of the superior cerebellar peduncle in *Ptf1a^Cre^;Vglut2^fx/fx^;ROSA^lsl-Arch/EYFP^* mice, behavioral video data was collected over four consecutive days. To establish a baseline for how much time the mice exhibit dystonic crises over a 35-minute session without optogenetic manipulation, we performed this experiment without any photostimulation on Day 0 (Figure 3B). To test whether photostimulation influenced the percentage of time spent in dystonic attack, video was recorded for five minutes before and five minutes during stimulation. To increase confidence that any behavioral change was a result of optogenetic manipulation, we repeated the stimulation paradigm three times on each day, resulting in a 35-minute session for each day (Figure 3B).

For unilateral photoactivation of the superior cerebellar peduncle and bilateral DBS of the CL in *Ptf1a^Cre^;Vglut2^fx/fx^;ROSA^lsl-Chr2/EYFP^* mice, we collected behavioral video data over four consecutive days. Each day consisted of an 86-minute session divided into five distinct periods: photobaseline, DBS period, immediately post-DBS, 30 minutes post-DBS, and 1 hour post-DBS (Figure 4G). During each distinct period, a 2-minute stimulation of blue light (470 nm light; 50 Hz square pulses; ≥ 1mW at fiber tip) was administered followed by 2 minutes of no light stimulation. This paradigm was repeated three times for each distinct time period. The percentage of time spent in an attack was quantified for each of the three light pulses, and the average of these values was analyzed. To establish a baseline for how much time the mice spend in a dystonic crisis induced by iCNN photoactivation, we performed this experiment without any DBS on Day 0 (Figure 4G). DBS was administered on Days 1-3. DBS was only performed in the DBS period for 10 minutes (130 Hz, 60us, 30uA), starting with the onset of the first light pulse and ending with the offset of the third light pulse.

### Tissue processing and immunohistochemistry

Perfusion and tissue fixation were performed as previously described (65). In brief, mice were anesthetized with Avertin (2, 2, 2-Tribromoethanol, Sigma-Aldrich, St. Louis, MO, USA; #T48402) via intraperitoneal injection. Once mice were deeply anesthetized, a whole-body perfusion was performed first with ice-chilled 0.1M phosphate-buffered saline (PBS; pH 7.4), then with ice-chilled 4% paraformaldehyde (4% PFA) diluted in PBS. The brain was then dissected out and placed in 4% PFA for 24 to 48 hr for post-fixation at 4°C. Cryoprotection was then performed by placing the tissue in stepwise sucrose dilutions, first in 15% sucrose in PBS followed by 30% sucrose in PBS. Brains were stored at 4°C during stepwise sucrose incubation steps. After cryoprotection, the tissue was embedded in Tissue-Tek O.C.T. Compound (Sakura, Torrance, CA, USA) and frozen at −80°C. For immunohistochemistry, free-floating sections cut at 40um on the cryostat were incubated in antibodies in a solution of 10% normal goat or donkey serum and 0.01% Tween-20. For all stains, sections were blocked for 2h with the blocking solution at room temperature and then incubated in primary antibodies overnight at room temperature. Sections were then washed with PBS 3 times for 5 min each and then incubated in secondary antibodies for 2 h. Sections were washed again 3 times in PBS for 5 min each and mounted onto slides. Slides with fluorescent signal were immediately cover-slipped using FluoroGel with Dapi as a medium. Antibodies used for immunohistochemistry (primary antibodies) were: GFP to visualize ChR2 and Sun1-GFP (Ch, Abcam, 1:1000), NeuroTrace fluorescent Nissl 530/615 to visualize neurons (Molecular Probes Inc, Eugene, OR, USA, #N21482; 1:1500), and Streptavidin Alexa Fluor 647 to visualize neurons filled with BDA 3K (Thermo Fisher Scientific, Eugene, OR, USA, #S21374); Secondary antibodies were: donkey anti-chicken secondary antibodies conjugated to 488 fluorophores (Invitrogen) diluted to 1:1500.

### *In situ* hybridization (ISH)

ISH was performed by the RNA ISH Core at Baylor College of Medicine using an automated robotic platform as described previously (66). ISH was used to visualize *Vglut2*, *Vgat*, and *YFP* expression in unfixed, fresh frozen tissue cut in 25 μm-thick coronal brain sections. Digoxigenin (DIG)-labeled mRNA antisense probes against *Vglut2*, *Vgat*, and *YFP* were generated using reverse-transcribed mouse cDNA as a template and an RNA DIG-labeling kit from Roche. Primer and probe sequences for the *Vglut2*, *Vgat*, and *YFP* probes are available on the Allen Brain Atlas website (http://www.brain-map.org).

### Imaging of immunostained tissue sections

Photomicrographs of stained tissue sections were captured using either a Zeiss Axio Imager.M2 microscope equipped with Zeiss AxioCam MRm and MRc5 cameras (Zeiss, Oberkochen, Germany) or a Leica DM4000 B LED microscope equipped with Leica DFC365 FX and Leica DMC 2900 cameras (Leica Microsystems Inc, Wetzlar, Germany). Zeiss Zen software was used for image acquisition from the Zeiss microscope. Leica Application Suite X (LAS X) software was used for image acquisition from the Leica microscope. Images were corrected for brightness and contrast using Adobe Photoshop CS5 (Adobe Systems, San Jose, CA, USA) for figure preparation. Schematics were made in Adobe Illustrator CC.

### Data analysis and statistics

Data are presented as mean±s.e.m. and were analyzed with a Wilcoxon matched-pairs signed rank test (non-normally distributed data with unequal variance). For all statistical tests, *P*<0.05 was considered as statistically significant. All statistical analyses were performed using GraphPad Prism (GraphPad Software, La Jolla, CA, USA) software.

## Supplementary Material

1

## Figures and Tables

**Table 1.** Clinical characteristics of patients with dystonic crisis, also known as status dystonicus (SD). Table outlines clinical characteristics of 30 patients admitted to Texas Children’s Hospital from 2015-2019 with acquired etiologies of dystonic crisis. Top grouping includes patient with only supratentorial findings, while bottom grouping includes those with cerebellar findings. Summarized imaging findings are as indicated in the respective columns. Ages are shown in years. “Dystonia phenomenology” refers to the description of dystonia based on consensus classifications while “Description of phenomenology” indicates the patient specific presentation of dystonia during SD. Patients with imaging depicted in figure 1 are as follows: # = top left, $ = bottom left, € = top middle, % = bottom middle, £ = top right, & = bottom right. Abbreviations of treatments with clinical benefit: BZ = benzodiazepine; α-2 = Alpha-2 agonists; AS = antispasmodic agents; ChemD = chemodenervation; VMAT2 = VMAT-2 inhibitor; None = no therapy with clinical benefit; Dopa = dopaminergic agonism; ITB = intrathecal baclofen pump; NmB = neuromuscular blockade.

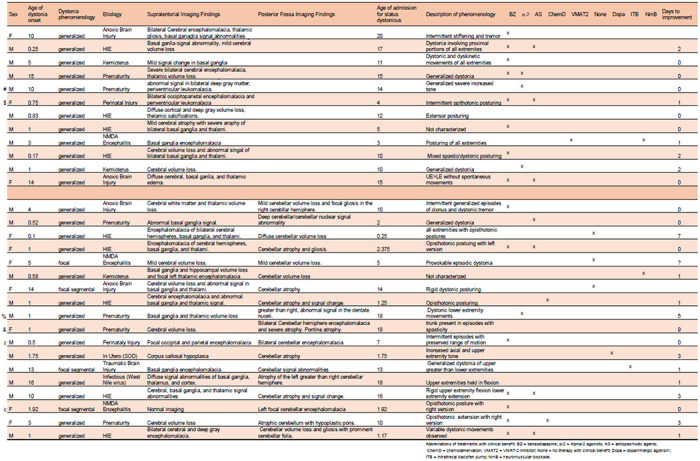

## Data Availability

The data that support the findings of this study are available from the corresponding author upon request.

## Data and materials availability:

All data, code, and materials are available in the main text, supplementary materials, or available upon request to the corresponding author.
