# Supplementary material for: Human and mouse cerebellar inhibitory circuits in dystonic crisis and their modulation with therapeutic stimulation
Source: bioRxiv. 2026 Apr 3:2026.04.01.715774. Preprint. [Version 1] doi: 10.64898/2026.04.01.715774 (PMC13060226; doi:10.64898/2026.04.01.715774)
Supplement: 1 [file NIHPP2026.04.01.715774v1-supplement-1.pdf]

bioRxiv preprint doi: https://doi.org/10.64898/2026.04.01.715774; this version posted April 3, 2026. The copyright holder for this preprint (which was not certified by peer review) is the author/funder, who has granted bioRxiv a license to display the preprint in perpetuity. It is made available under aCC-BY 4.0 International license.

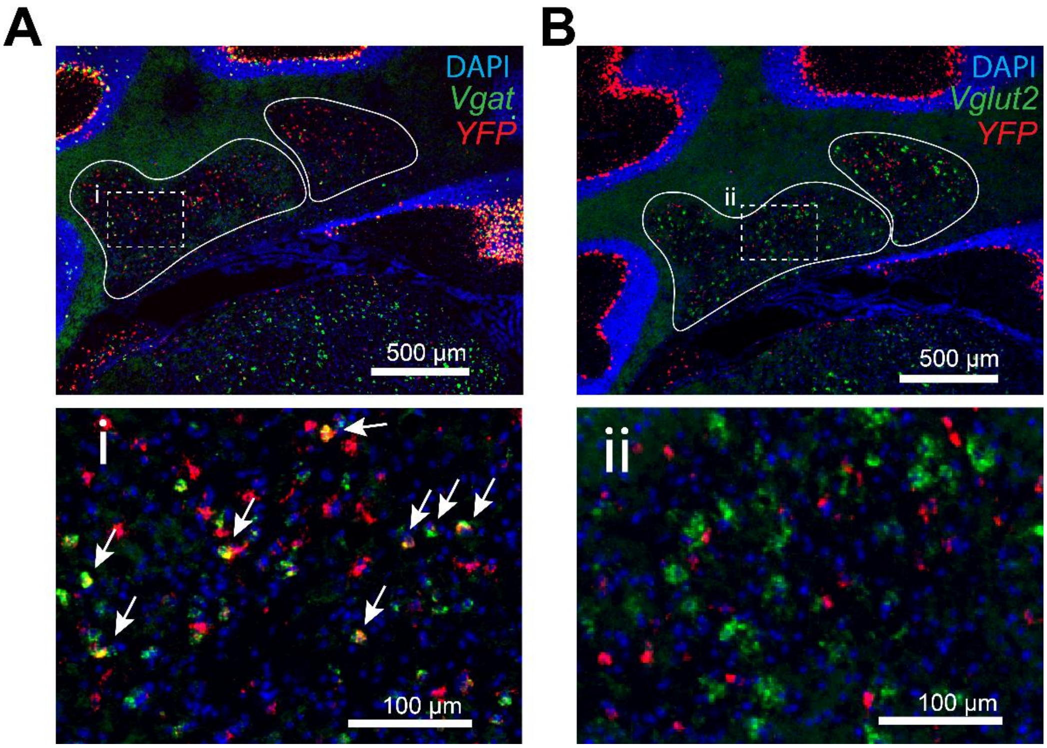

Supplemental Figure 1: **Ptf1a-cre induced expression of cre-dependent ChR2-YFP in inhibitory cerebellar nuclei neurons, but not excitatory cerebellar nuclei neurons, in *Ptf1a^{Cre};Vglut2$^{fx/fx}$;ROSA$^{lsl\text{-}Chr2/EYFP}$* mice**. (**A**) *In situ* hybridization in a coronal section showing mRNA expression *Vgat* and *YFP* as well as DAPI staining (n=3 animals; 3 sections per animal). The cerebellar nuclei are outlined in white. Inset (i) is a higher magnification image from (**A**) with white arrows marking cerebellar nuclei neurons that were co-labeled with *Vgat* and *YFP* probes. (**B**) *In situ* hybridization in a coronal section showing mRNA expression *Vglut2* and *YFP* as well as DAPI, with the cerebellar nuclei outlined in white (n=3 animals; 3 sections per animal). Inset (ii) is a higher magnification image of (**B**) showing that there was no co-labeling of *Vgut2* and *YFP*.

bioRxiv preprint doi: https://doi.org/10.64898/2026.04.01.715774; this version posted April 3, 2026. The copyright holder for this preprint (which was not certified by peer review) is the author/funder, who has granted bioRxiv a license to display the preprint in perpetuity. It is made available under aCC-BY 4.0 International license.

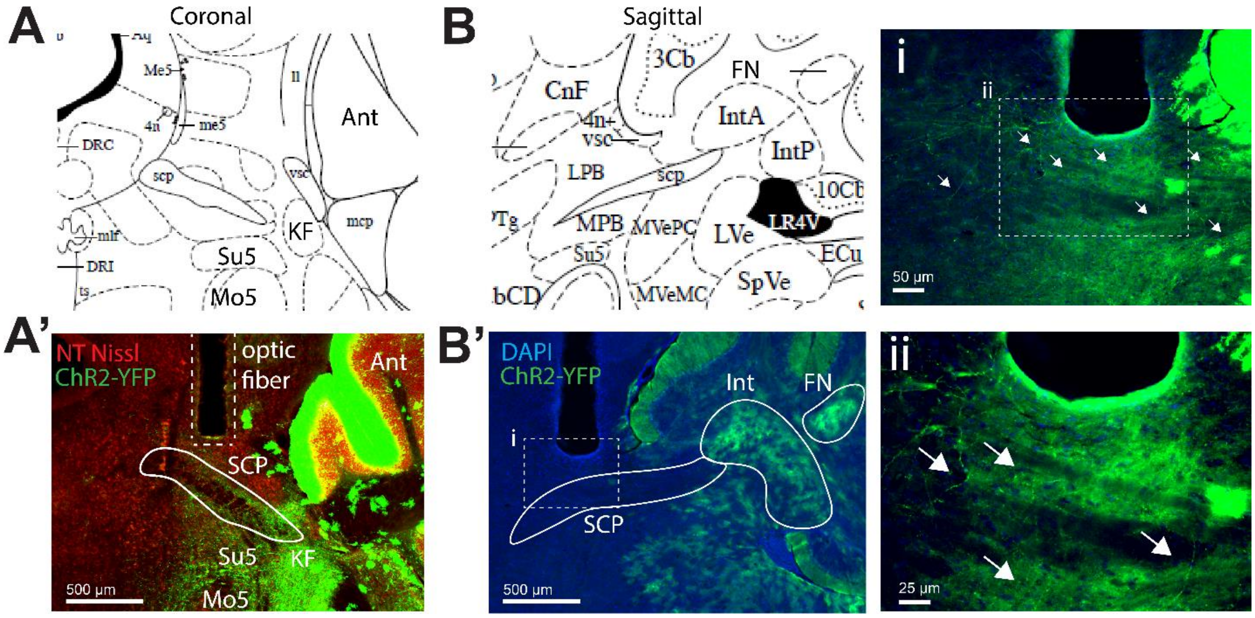

Supplemental Figure 2: Anatomical verification of optical fibers over the superior cerebellar peduncle (SCP), which houses extracerebellar projections from inhibitory cerebellar nuclei neurons as labeled with ChR2-YFP in *Ptf1a*^{Cre}*;Vglut2*$^{fx/fx}$*;ROSA*$^{lsl\text{-}Chr2/EYFP}$ mice. (**A**) Schematic from Paxinos and Franklin of a coronal section showing the stereotaxic location of the SCP used for targeting the optical fibers as verified in an immunohistochemical stain in (**A’**; n=9 animals, 3 sections per animal). Note the punctate labeling of axonal projections expressing ChR2-YFP in the SCP. (**B**) Schematic from Paxinos and Franklin of a sagittal section showing the stereotaxic location of the SCP used for targeting the optical fibers as verified in (**B’**; n=3 animals, 3 sections per animal). Insets (i and ii) show higher magnification of the SCP from (**B’**), highlighting the expression of ChR2-YFP in axonal projections with white arrows. SCP, superior cerebellar peduncle; Ant, anterior cerebellar lobe; KF, Kolliker-Fuse nucleus; Su5, supratrigeminal nucleus; Mo5, trigeminal motor nucleus; Int, interposed cerebellar nucleus; FN, fastigial cerebellar nucleus.

bioRxiv preprint doi: https://doi.org/10.64898/2026.04.01.715774; this version posted April 3, 2026. The copyright holder for this preprint (which was not certified by peer review) is the author/funder, who has granted bioRxiv a license to display the preprint in perpetuity. It is made available under aCC-BY 4.0 International license.

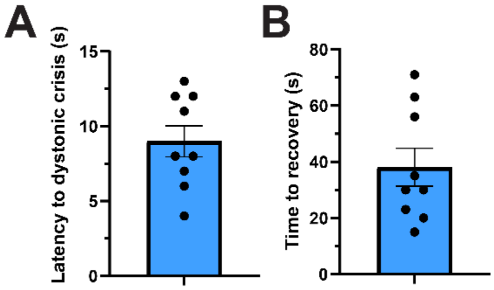

Supplemental Figure 3: Photoactivation of the inhibitory cerebellar nuclei neuron pathway through the superior cerebellar peduncle induced dystonic crises that occurred within a short latency from light onset and that persisted many seconds after stimulation ends in *Ptf1a*^{Cre}*;Vglut2*$^{fx/fx}$*;ROSA*$^{lsl\text{-}Chr2/EYFP}$ mice. (**A**) Dystonic crises were induced at around 10 seconds from light onset. (**B**) After the light stimulation ends, the induced dystonic crisis persisted for around 40 seconds. n=9 animals.

bioRxiv preprint doi: https://doi.org/10.64898/2026.04.01.715774; this version posted April 3, 2026. The copyright holder for this preprint (which was not certified by peer review) is the author/funder, who has granted bioRxiv a license to display the preprint in perpetuity. It is made available under aCC-BY 4.0 International license.

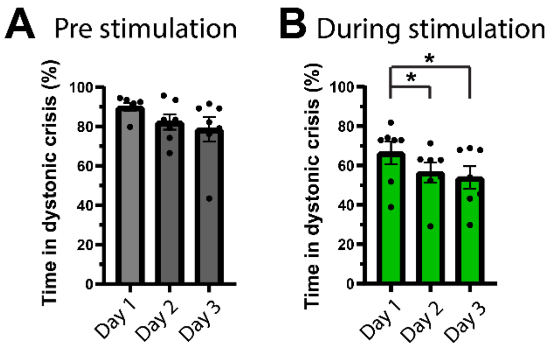

Supplemental Figure 4: Photostimulation over several days resulted in a reduction in the time spent in dystonic crisis. (**A**) For the pre stimulation periods when comparing Day 1 (the first day of stimulation) against Day 2 (p=0.0625) and Day 3 (p=0.0781), there was a trend toward a reduction in dystonic crisis, but it was not statistically significant. (**B**) For the during stimulation periods, there was a significant reduction in dystonic crises when comparing Day 1 to Day 2 (p=0.0312) and Day 3 (p=0.0156). For all statistics, n=7 animals, Wilcoxon matched-pairs signed rank test, two tailed.

bioRxiv preprint doi: https://doi.org/10.64898/2026.04.01.715774; this version posted April 3, 2026. The copyright holder for this preprint (which was not certified by peer review) is the author/funder, who has granted bioRxiv a license to display the preprint in perpetuity. It is made available under aCC-BY 4.0 International license.

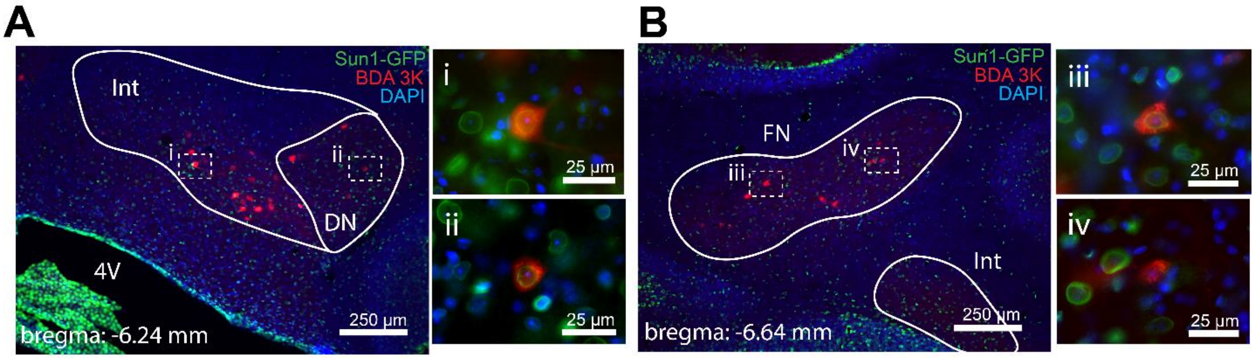

Supplemental Figure 5: Inhibitory cerebellar nuclei neurons from all three major cerebellar nuclei (fastigial, interposed, dentate) project to the centrolateral nucleus of the thalamus (n=4 animals, 3 sections per animal). (**A**) Coronal section showing that neurons from the interposed and dentate cerebellar nucleus were filled with BDA 3K following injection of BDA 3K, a predominantly retrograde tracer, in the centrolateral nucleus of the thalamus. Insets (i) and (ii) are higher magnification images of (**A**) showing the co-labeling of DAPI, Sun1-GFP, and BDA 3K in the interposed and dentate cerebellar nucleus, respectively. (**B**) Coronal section showing that neurons from the fastigial cerebellar nucleus were filled with BDA 3K following injection of BDA 3K, a predominantly retrograde tracer, in the centrolateral nucleus of the thalamus. Inset (iii) is a higher magnification image of (**B**) showing a co-labeling of DAPI, Sun1-GFP, and BDA 3K. Inset (iv) is a higher magnification image of (**B**) showing co-labeling of DAPI and BDA 3K, but not Sun1-GFP. Int, interposed cerebellar nucleus; DN, dentate cerebellar nucleus; FN, fastigial cerebellar nucleus; 4V, fourth ventricle.

bioRxiv preprint doi: https://doi.org/10.64898/2026.04.01.715774; this version posted April 3, 2026. The copyright holder for this preprint (which was not certified by peer review) is the author/funder, who has granted bioRxiv a license to display the preprint in perpetuity. It is made available under aCC-BY 4.0 International license.

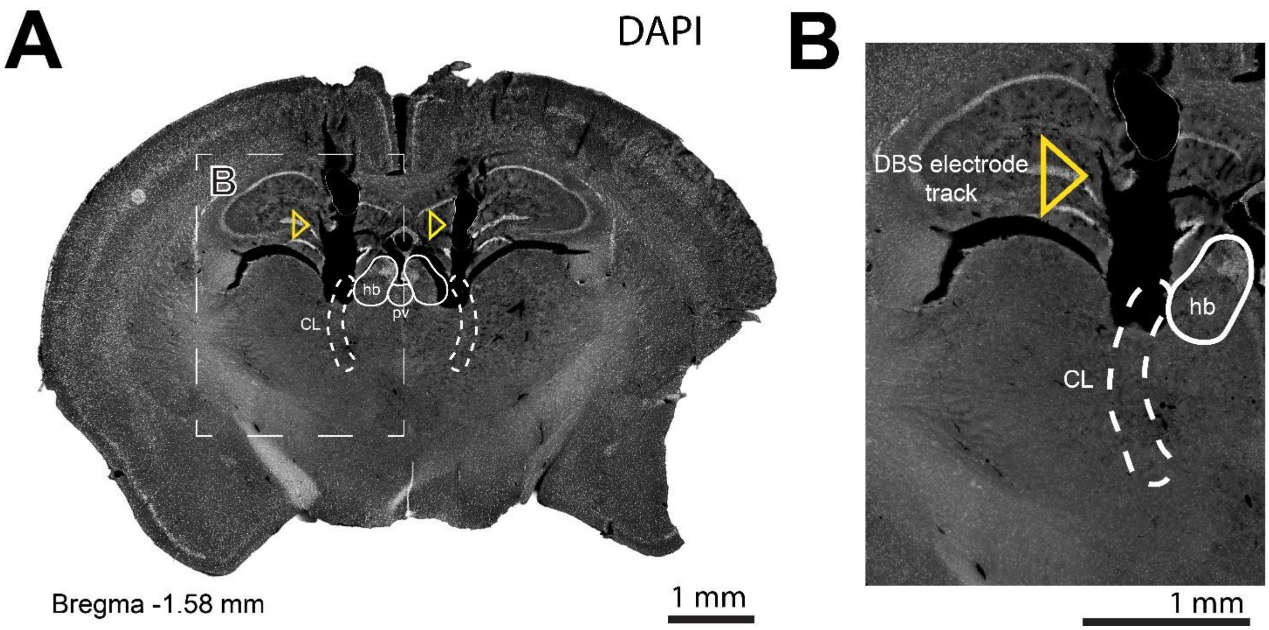

Supplemental Figure 6: Anatomical verification of the surgical targeting of deep brain stimulation electrodes in the centrolateral nucleus of the thalamus. (**A**) Immunohistochemical staining of DAPI showing the tracks formed by the bilateral implantation of deep brain stimulation electrodes in the centrolateral nucleus of the thalamus (n=6 animals, 3 sections per animal). Yellow triangles mark the tracks formed by the DBS electrodes above and with some penetration into the centrolateral nucleus of the thalamus. (**B**) Inset of A showing a higher magnified image of the DBS electrode track in the centrolateral nucleus of the thalamus. CL, centrolateral nucleus of the thalamus; hb, habenula; pv, paraventricular thalamic nucleus.

bioRxiv preprint doi: https://doi.org/10.64898/2026.04.01.715774; this version posted April 3, 2026. The copyright holder for this preprint (which was not certified by peer review) is the author/funder, who has granted bioRxiv a license to display the preprint in perpetuity. It is made available under aCC-BY 4.0 International license.

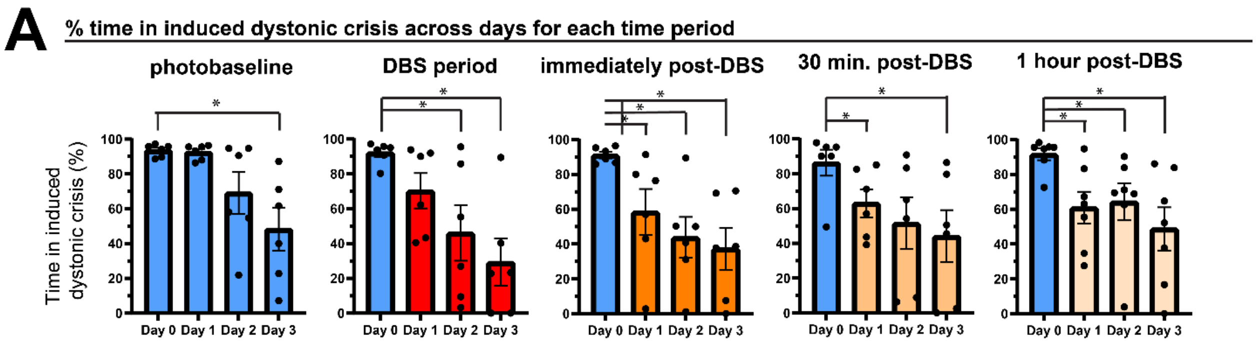

Supplemental Figure 7: Deep brain stimulation of the centrolateral nucleus of the thalamus over several days reduced the time in dystonic crises induced by photoactivation of the inhibitory cerebellar nuclei neuron pathway. (**A**) Comparing the induced dystonic crises during photobaseline on Day 0 against Day 3 revealed a significant reduction in dystonic crises (p=0.0312). Photoinduced dystonic crises during DBS on Day 0 were reduced on Day 2 (p=0.0312) and Day 3 (p=0.0312). Photoinduced dystonic crises immediately post-DBS on Day 0 were decreased on Day 1 (p=0.0312), Day 2 (p=0.0312), and Day 3 (p=0.0312). For 30 minutes post-DBS, photoinduced crises were reduced on Day 1 (p=0.0312) and Day 3 (p=0.0312). For 1 hour post-DBS, photoinduced crises decreased on Day 1 (p=0.0156), Day 2 (p=0.0156), and Day 3 (p=0.0156). For all statistics: n=6 animals, Wilcoxon matched-pairs signed rank test, two-tailed, error bars are defined as standard error of the mean.

bioRxiv preprint doi: https://doi.org/10.64898/2026.04.01.715774; this version posted April 3, 2026. The copyright holder for this preprint (which was not certified by peer review) is the author/funder, who has granted bioRxiv a license to display the preprint in perpetuity. It is made available under aCC-BY 4.0 International license.

Supplementary Movie 1: Movie of a patient in dystonic crisis at the hospital that was included in the study. The patient exhibits the debilitating dystonic postures characteristic of dystonic crisis including rigidity and stiffness in the neck, arms, fingers, and toes.

Supplementary Movie 2: Movie of a *Ptf1a*^{Cre}*;Vglut2*$^{fx/fx}$*;ROSA*$^{lsl\text{-}Chr2/EYFP}$ mouse before, during, and after photoactivation of the inhibitory cerebellar nuclei neuron pathway via the superior cerebellar peduncle. A dystonic crisis was induced within several seconds of light onset, and the crisis persisted for many seconds after the end of light stimulation.

Supplementary Movie 3: Movie of a *Ptf1a*^{Cre}*;ROSA*$^{lsl\text{-}Chr2/EYFP}$ mouse before, during, and after photoactivation of the inhibitory cerebellar nuclei neuron pathway via the superior cerebellar peduncle. Although no dystonic crisis was induced, the animal moved abnormally with slowed movement, mild dystonia-like muscle tension, crawling, widened hind legs, dragging of the trunk, clunky locomotion, and wide steps.

Supplementary Movie 4: Movie of a *Ptf1a*^{Cre}*;Vglut2*$^{fx/fx}$*;ROSA*$^{lsl\text{-}Arch/EYFP}$ mouse before and during photoinhibition of the inhibitory cerebellar nuclei neuron pathway via the superior cerebellar peduncle on Day 1 and Day 3 of photostimulation. The first half of the video consisted of footage from Day 1 of stimulation where there was a notable alleviation of the spontaneous dystonic crisis and restoration of mobility. On Day 3 of stimulation as shown in the second half of the video, the baseline time spent in spontaneous dystonic crisis was slightly less severe than Day 1, and photostimulation further improved the mobility of the dystonic mouse to a level that was better than that seen on Day 1 during photostimulation.

Supplementary Movie 5: Movie of a *Ptf1a*^{Cre}*;Vglut2*$^{fx/fx}$*;ROSA*$^{lsl\text{-}Chr2/EYFP}$ mouse on Day 1 and Day 3 of deep brain stimulation (DBS) in the centrolateral nucleus of the thalamus (CL) showing the short-term and long-term therapeutic effects of DBS on dystonic crises induced by iCNN photoactivation. On Day 1, the photobaseline shows that photoactivation of the iCNN pathway via the superior cerebellar peduncle reliably induced a dystonic crisis; however, when DBS of the CL was administered concurrently with the light stimulation in the "during DBS" period, a dystonic crisis was induced only briefly and then the mouse quickly recovered its mobility. On Day 3, the photobaseline period shows that iCNN photoactivation still reliably induced dystonic crisis; however, concurrent DBS of the CL in the "during DBS" period prevented the occurrence of a dystonic crisis induced by iCNN photoactivation.
